# Supplementary material for: Reciprocal regulation between the molecular clock and kidney injury
Source: Life Sci Alliance. 2023 Jul 24;6(10):e202201886. doi: 10.26508/lsa.202201886 (PMC10366531; doi:10.26508/lsa.202201886)
Supplement: Supplementary file 2 [file LSA-2022-01886_SdataF2_F3_F6_FS3_FS5_FS6_FS7.pptx]

## Slide 1
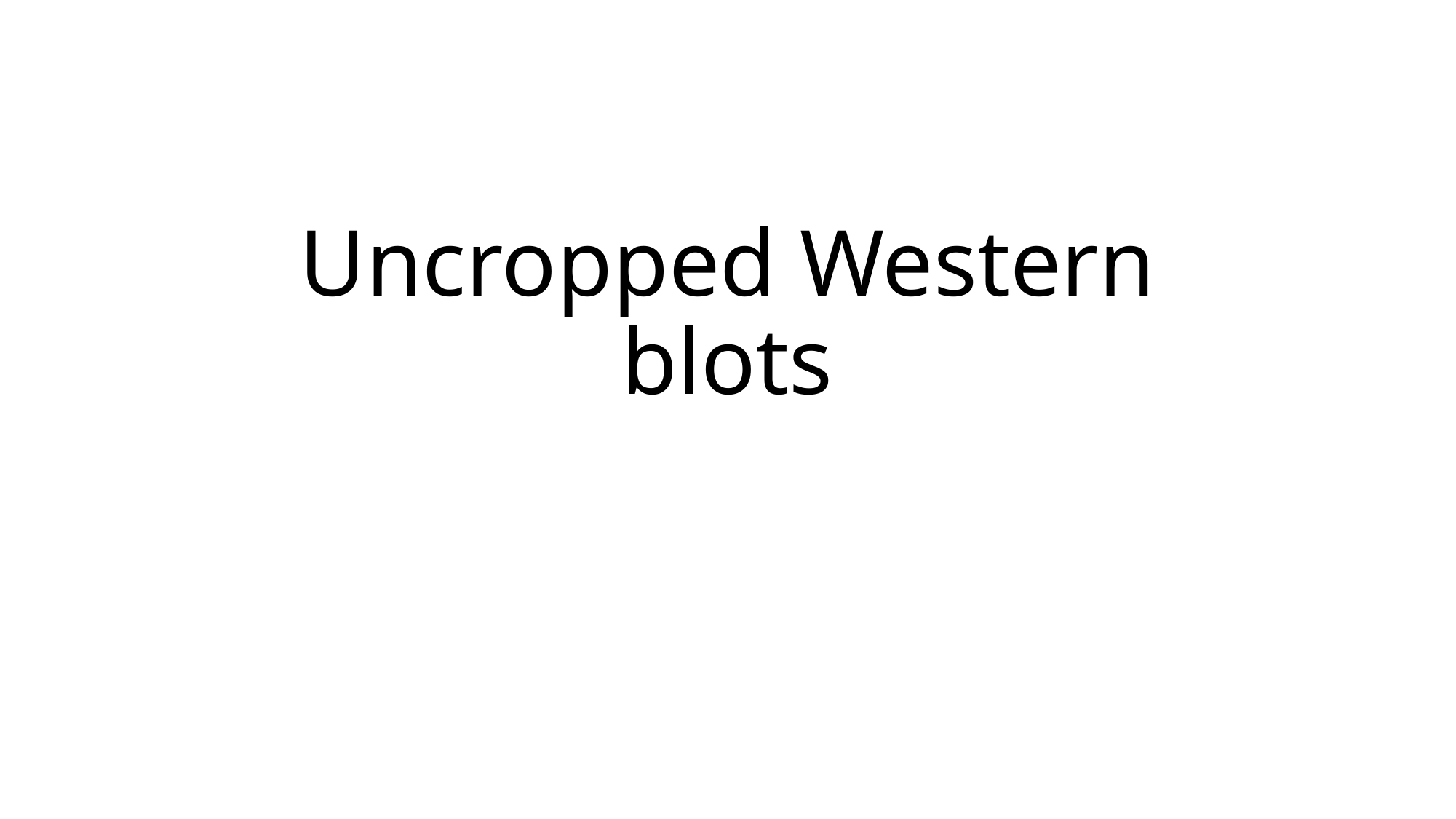

# Uncropped Western blots

## Slide 2
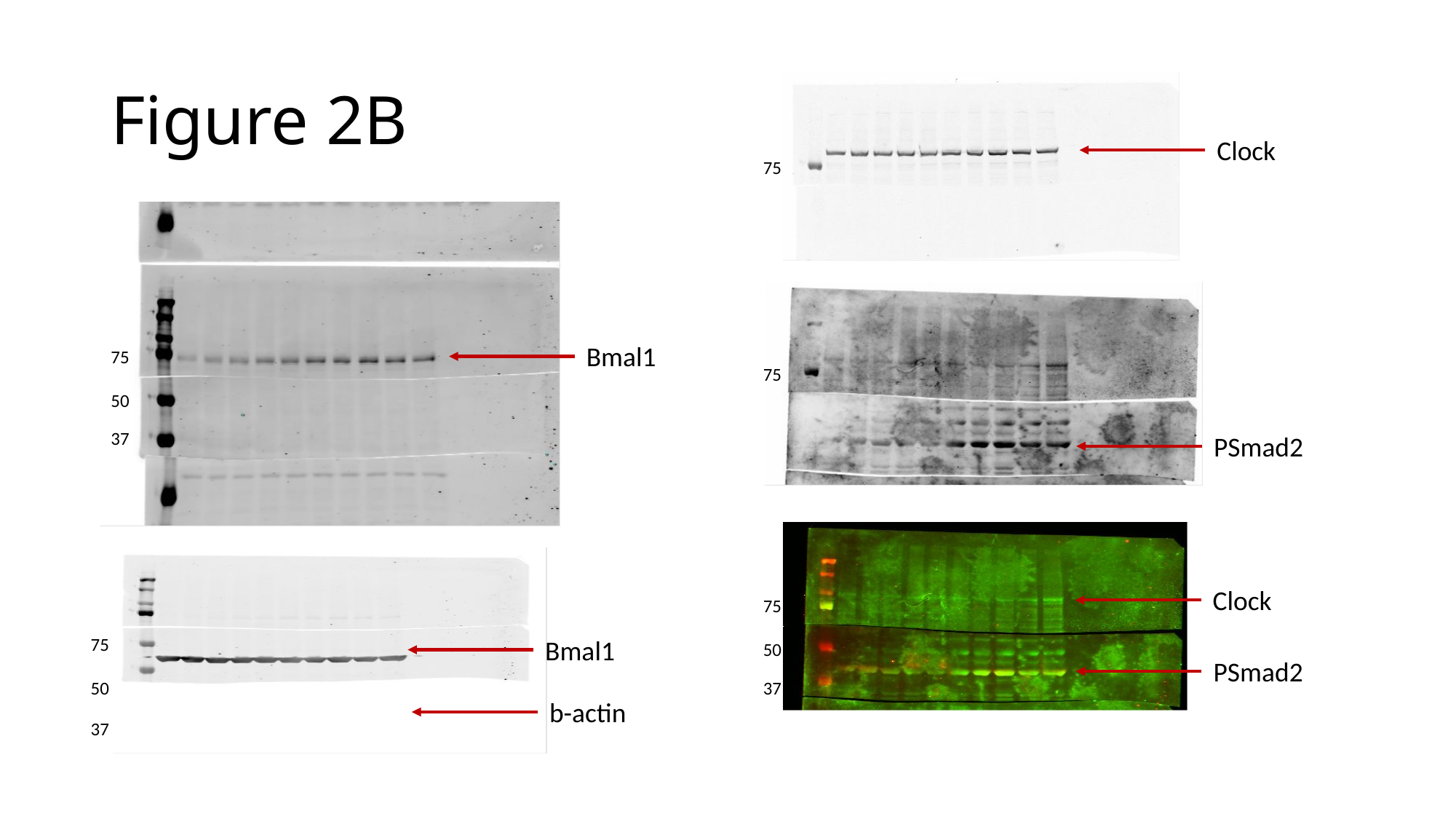

# Figure 2B
Clock
75
Bmal1
75
75
50
37
PSmad2
Clock
75
75
Bmal1
50
PSmad2
37
50
b-actin
37

## Slide 3
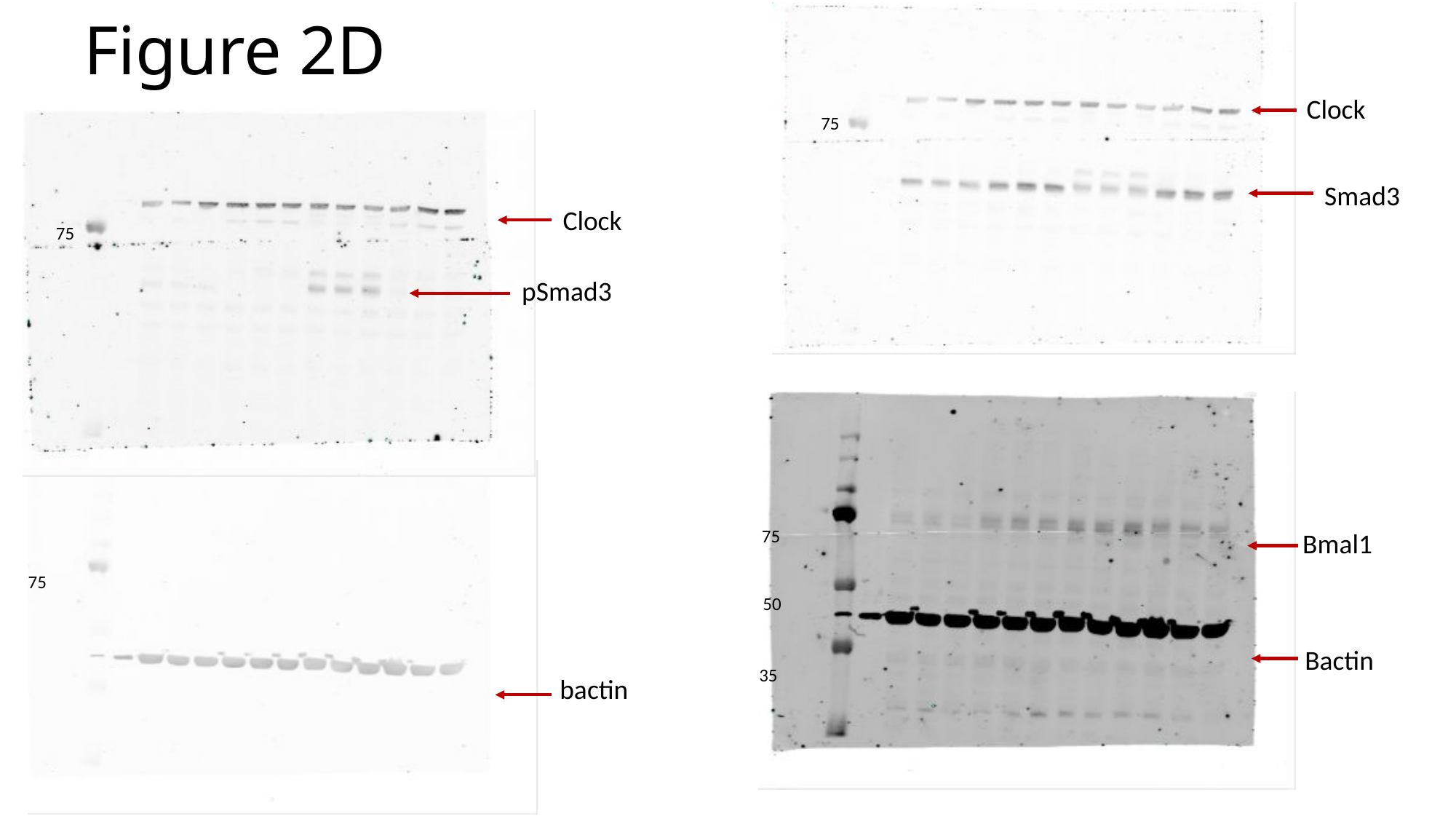

# Figure 2D
Clock
75
Smad3
Clock
75
pSmad3
75
Bmal1
75
50
Bactin
35
bactin

## Slide 4
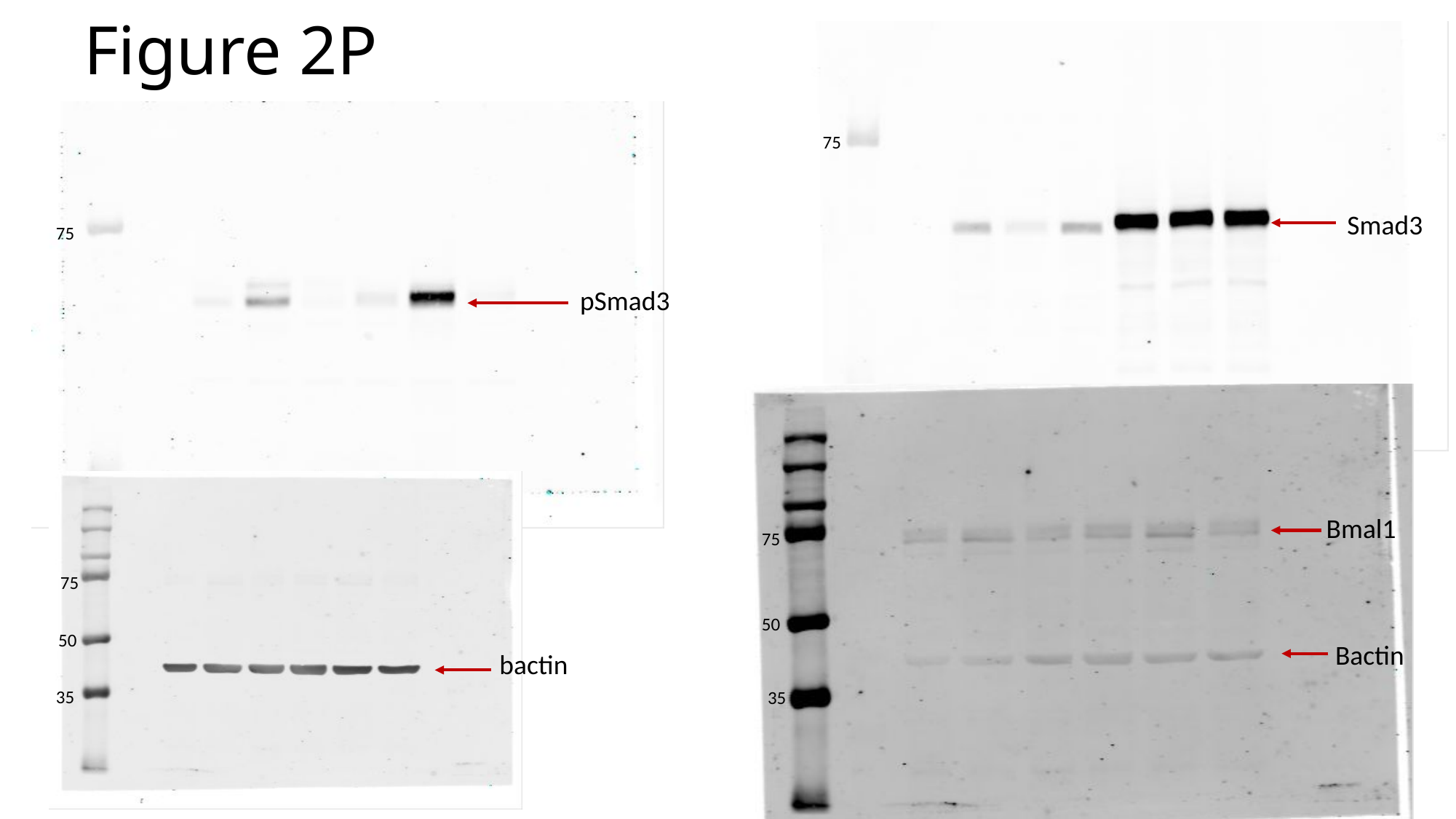

# Figure 2P
75
Smad3
75
pSmad3
Bmal1
75
75
50
50
Bactin
bactin
35
35

## Slide 5
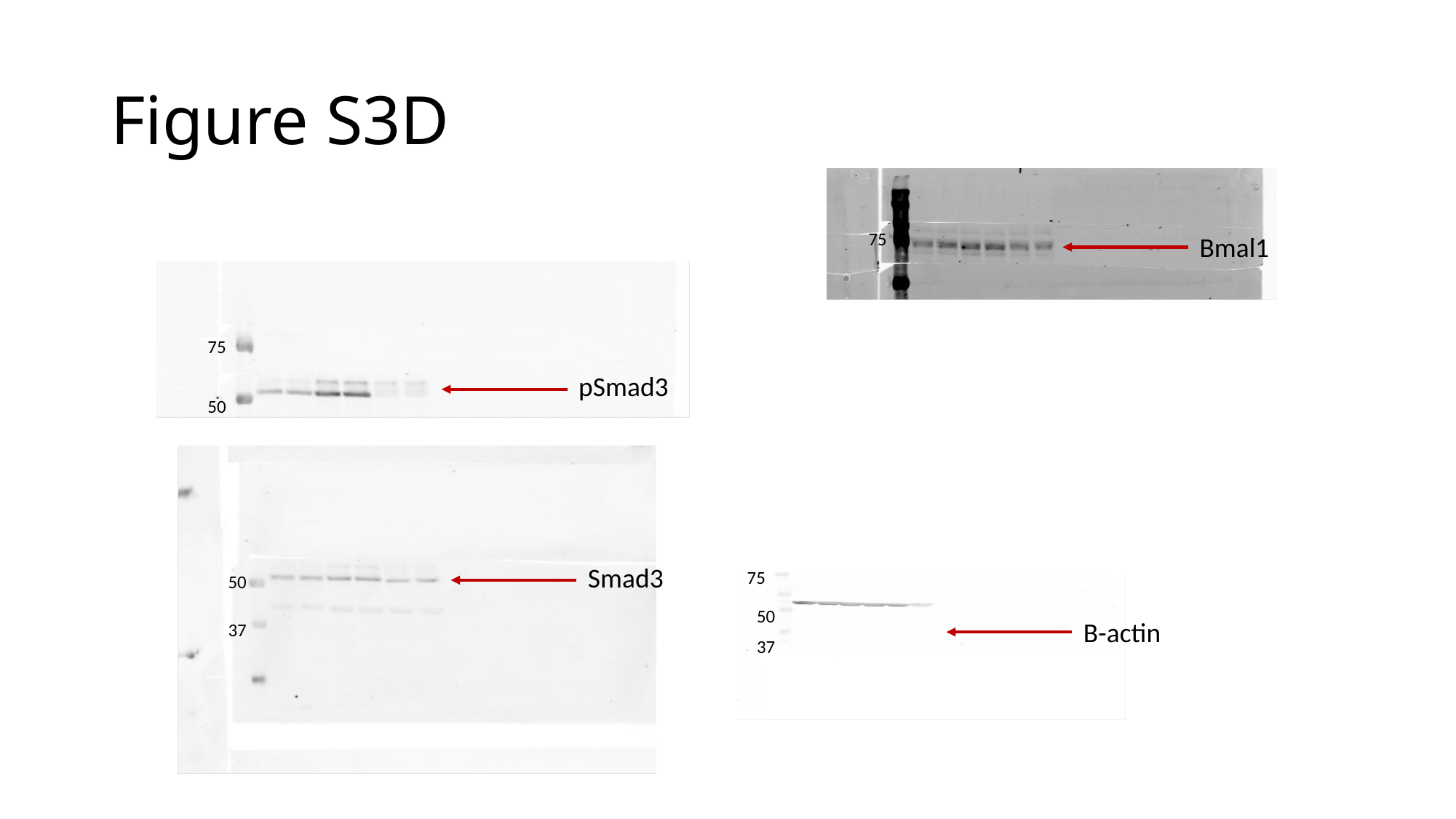

# Figure S3D
75
Bmal1
75
pSmad3
50
Smad3
75
50
50
B-actin
37
37

## Slide 6
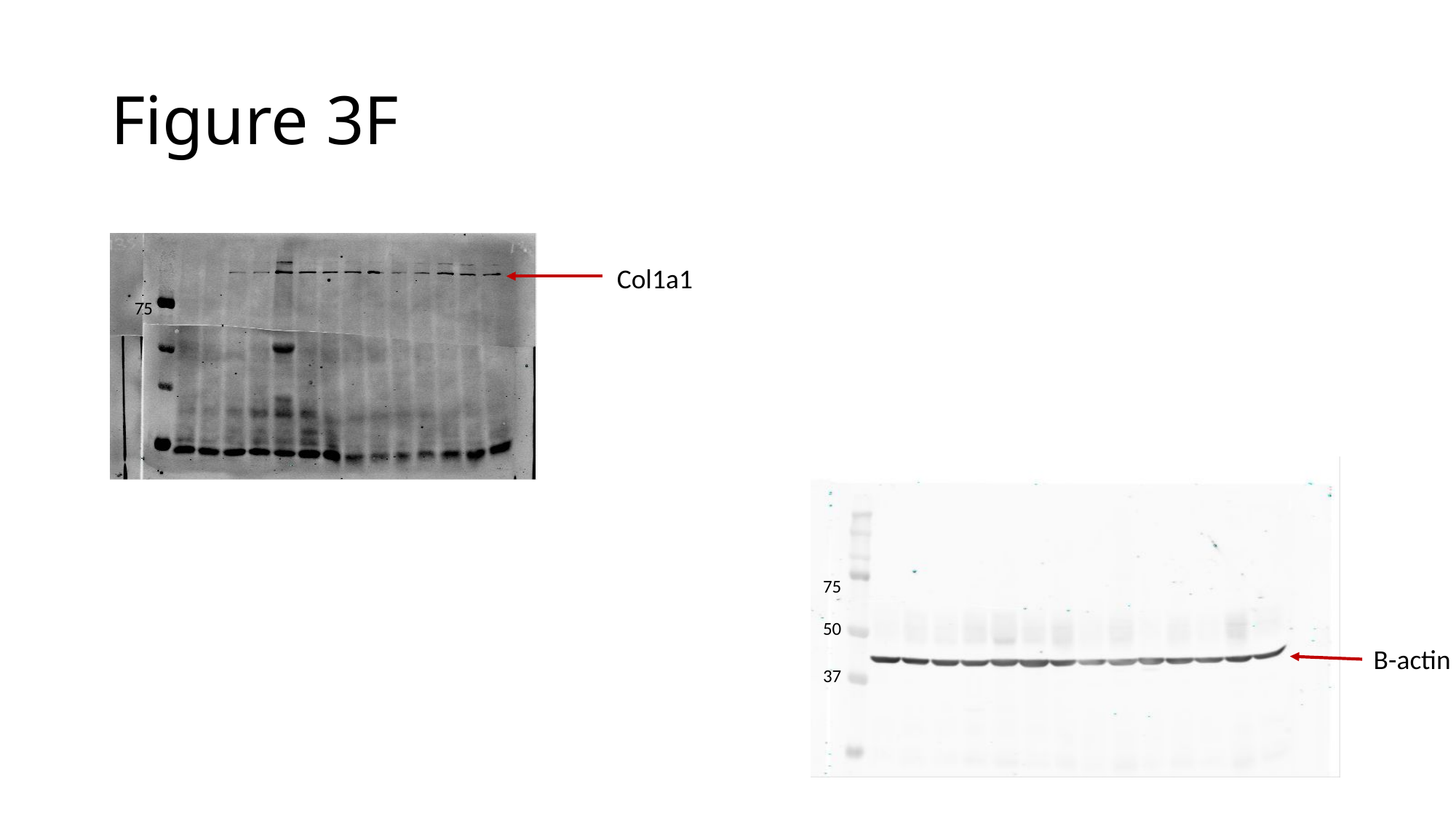

# Figure 3F
Col1a1
75
75
50
B-actin
37

## Slide 7
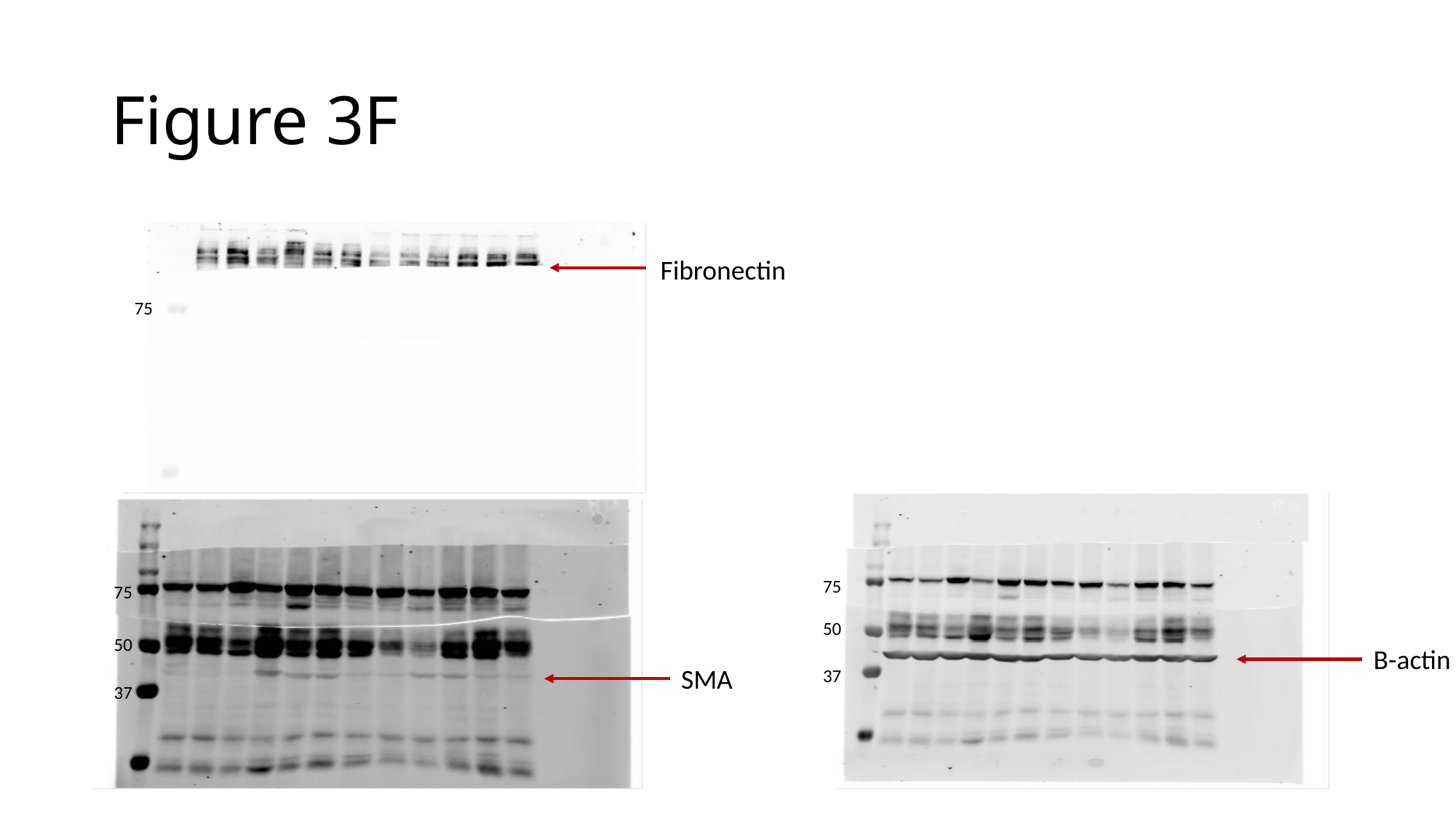

# Figure 3F
Fibronectin
75
75
75
50
50
B-actin
SMA
37
37

## Slide 8
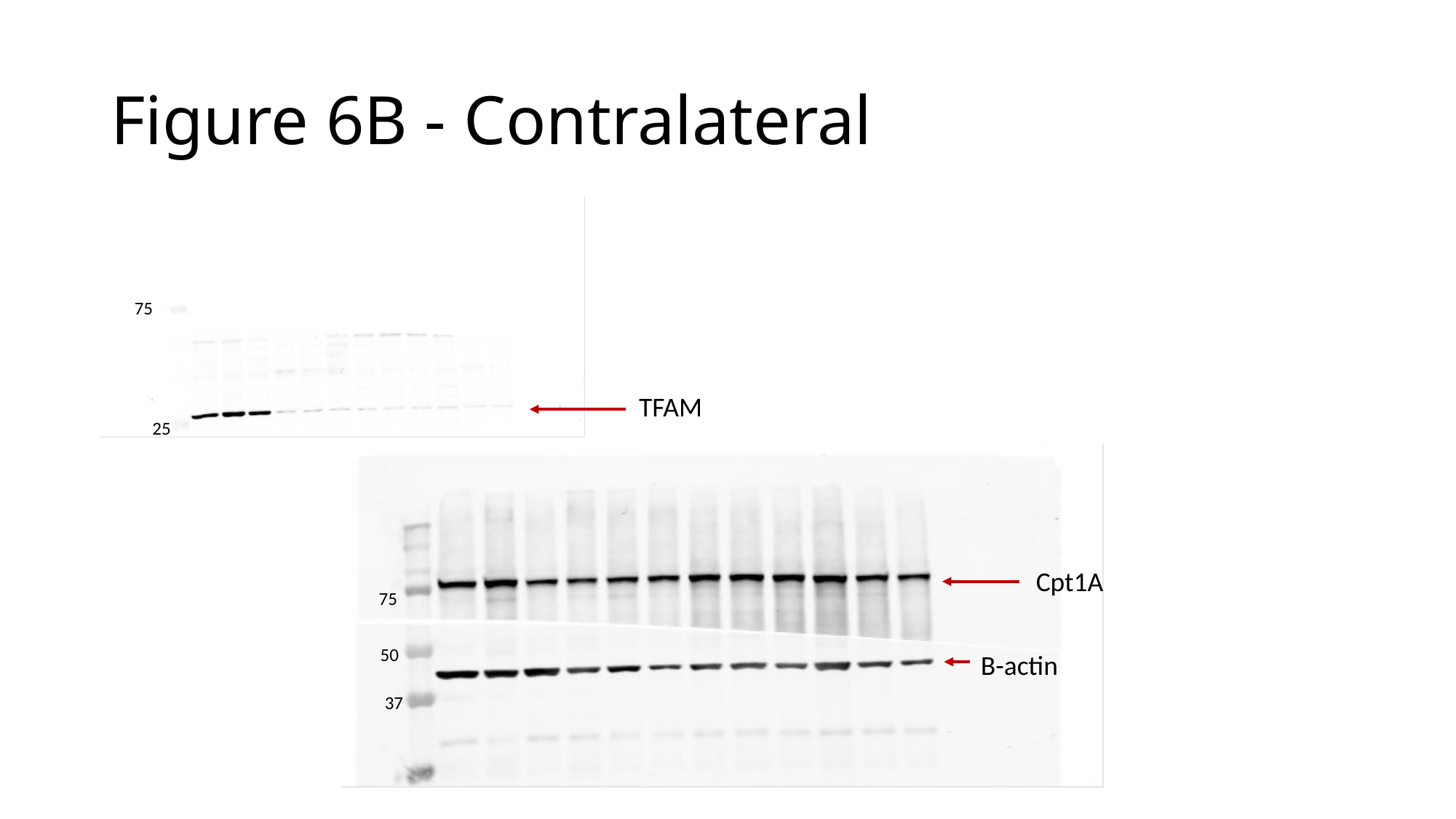

# Figure 6B - Contralateral
75
TFAM
25
Cpt1A
75
50
B-actin
37

## Slide 9
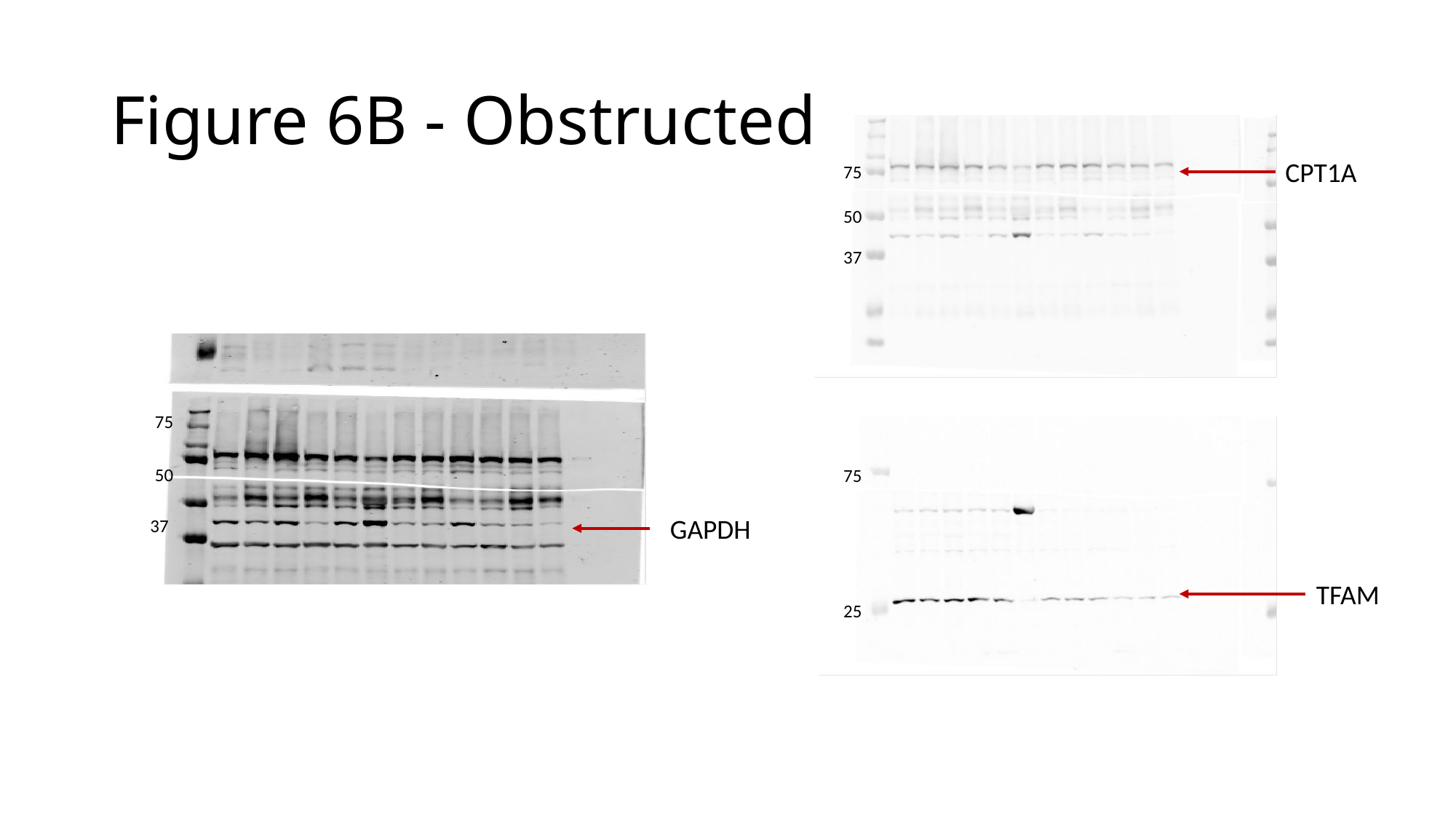

# Figure 6B - Obstructed
CPT1A
75
50
37
75
50
75
GAPDH
37
TFAM
25

## Slide 10
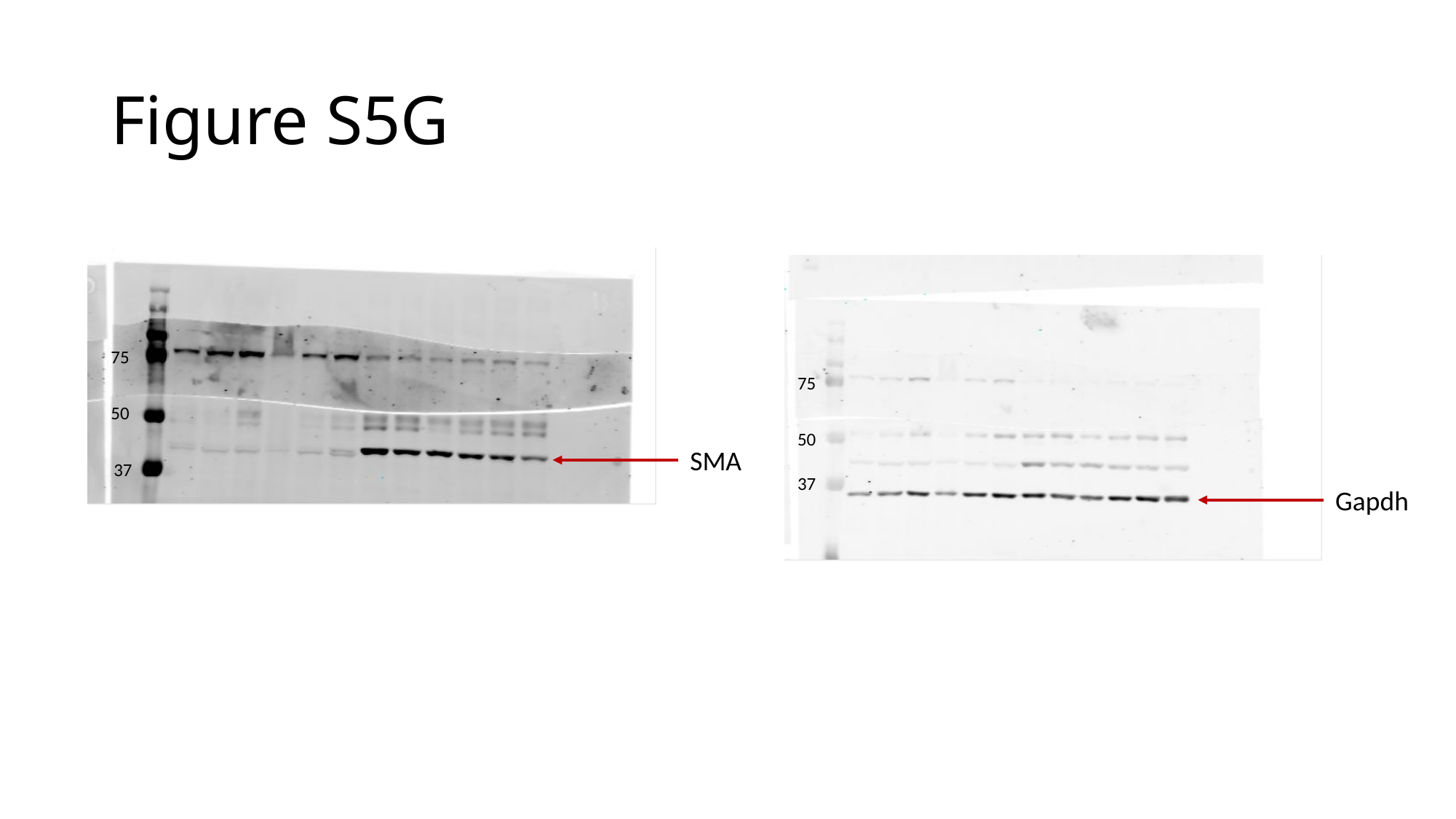

# Figure S5G
75
75
50
50
SMA
37
37
Gapdh

## Slide 11
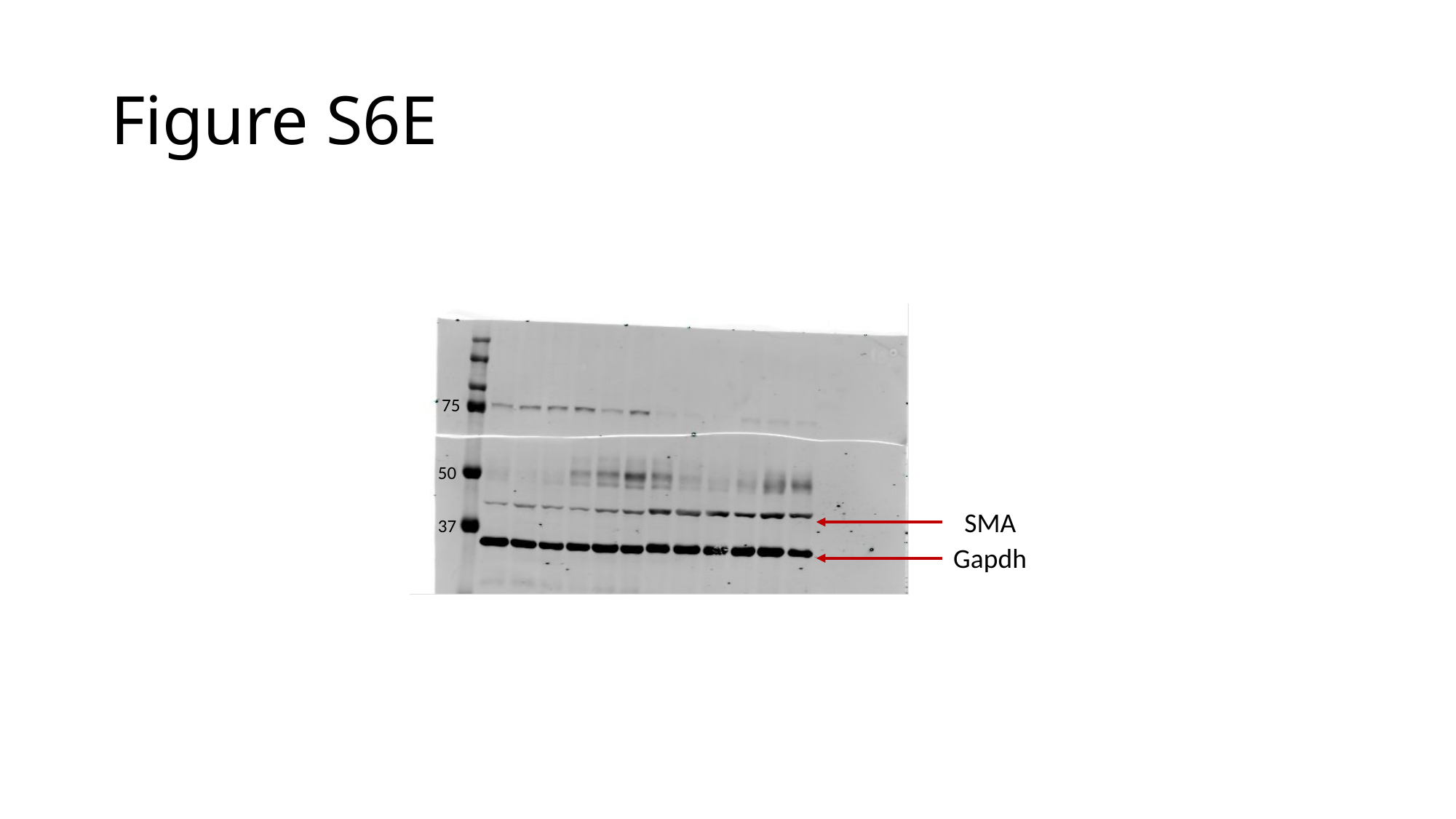

# Figure S6E
75
50
SMA
37
Gapdh

## Slide 12
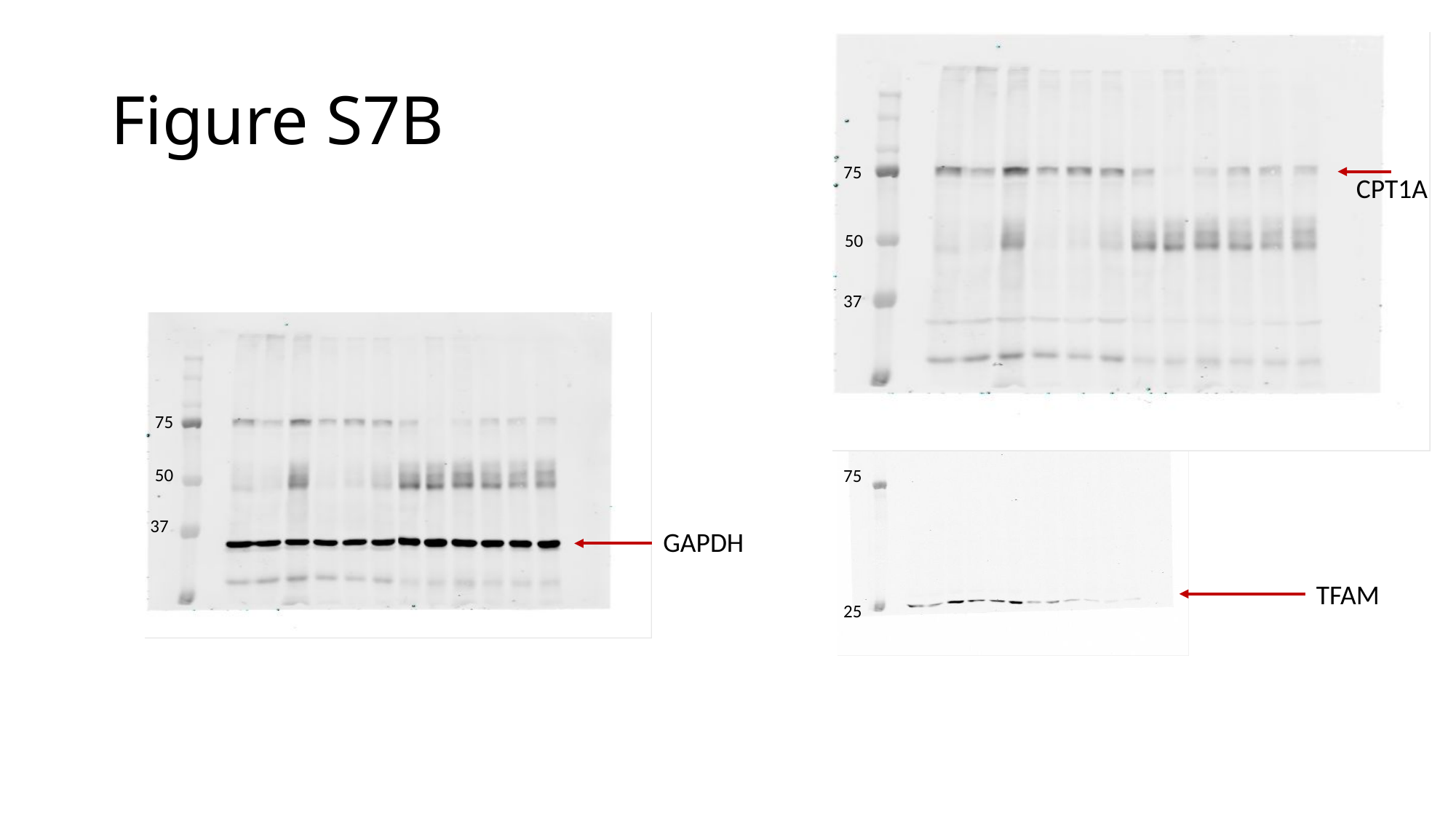

# Figure S7B
75
CPT1A
50
37
75
50
75
37
GAPDH
TFAM
25

## Slide 13
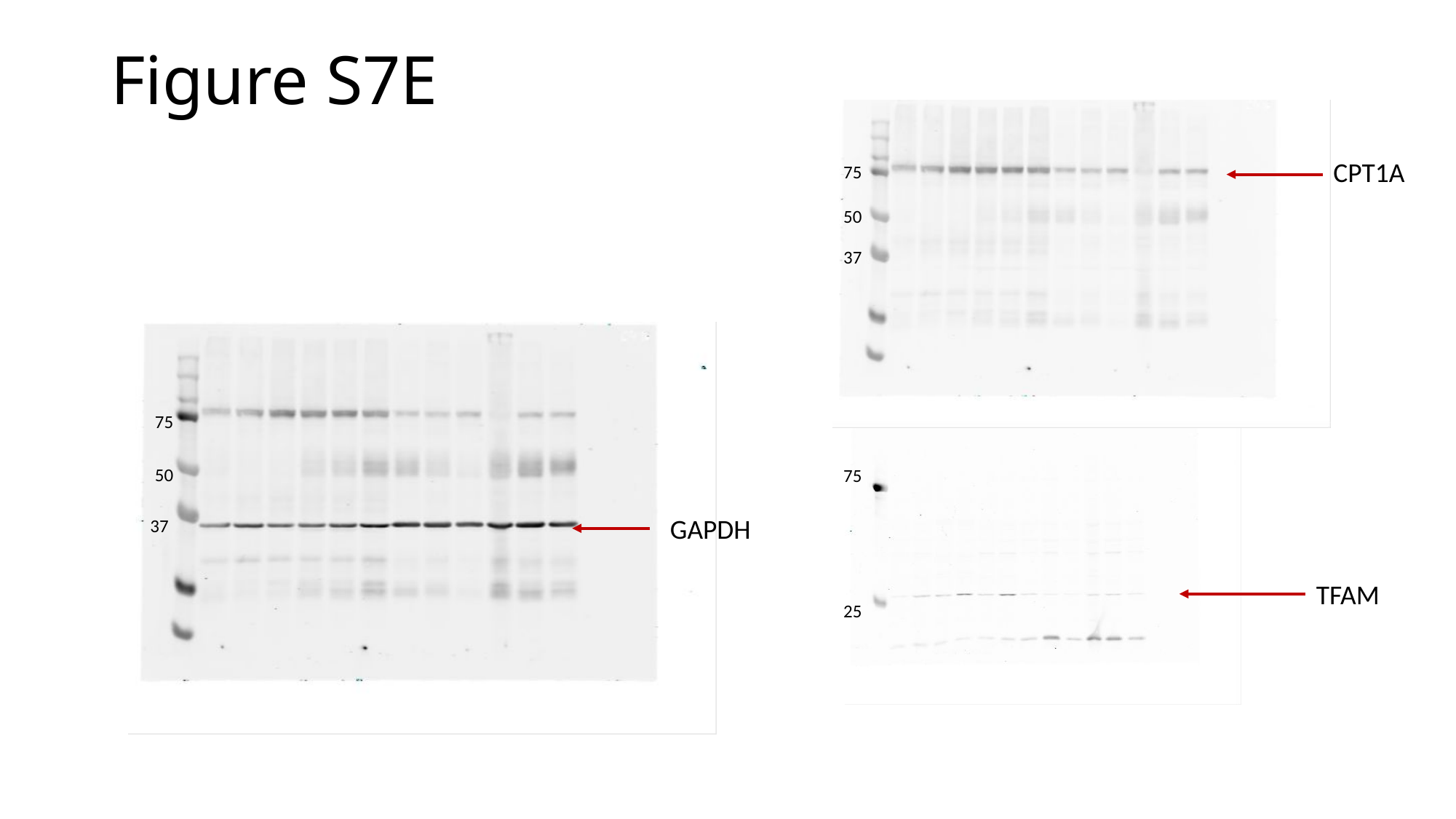

# Figure S7E
CPT1A
75
50
37
75
50
75
GAPDH
37
TFAM
25
